# 3-Dimensional Facial Analysis—Facing Precision Public Health

**DOI:** 10.3389/fpubh.2017.00031

**Published:** 2017-04-10

**Authors:** Gareth Baynam, Alicia Bauskis, Nicholas Pachter, Lyn Schofield, Hedwig Verhoef, Richard L. Palmer, Stefanie Kung, Petra Helmholz, Michael Ridout, Caroline E. Walker, Anne Hawkins, Jack Goldblatt, Tarun S. Weeramanthri, Hugh J. S. Dawkins, Caron M. Molster

**Affiliations:** ^1^Genetic Services of Western Australia, Department of Health, Government of Western Australia, Perth, WA, Australia; ^2^Western Australian Register of Developmental Anomalies, Perth, WA, Australia; ^3^Office of Population Health Genomics, Public Health Division, Department of Health, Government of Western Australia, Perth, WA, Australia; ^4^School of Paediatrics and Child Health, University of Western Australia, Perth, WA, Australia; ^5^Institute for Immunology and Infectious Diseases, Murdoch University, Perth, WA, Australia; ^6^Telethon Kids Institute, Perth, WA, Australia; ^7^Spatial Sciences, Department of Science and Engineering, Curtin University, Perth, WA, Australia; ^8^School of Pathology and Laboratory Medicine, University of Western Australia, Perth, WA, Australia; ^9^Centre for Comparative Genomics, Murdoch University, Perth, WA, Australia; ^10^Cooperative Research Centre for Spatial Information, Perth, WA, Australia; ^11^School of Spatial Sciences, Curtin University, Perth, WA, Australia; ^12^Public Health Division, Department of Health, Government of Western Australia, Perth, WA, Australia; ^13^Centre for Population Health Research, Curtin Health Innovation Research Institute, Curtin University of Technology, Perth, WA, Australia

**Keywords:** public health, 3D facial scan, rare diseases, spatial information, genomics and genetics, developmental disabilities

## Abstract

Precision public health is a new field driven by technological advances that enable more precise descriptions and analyses of individuals and population groups, with a view to improving the overall health of populations. This promises to lead to more precise clinical and public health practices, across the continuum of prevention, screening, diagnosis, and treatment. A phenotype is the set of observable characteristics of an individual resulting from the interaction of a genotype with the environment. Precision (deep) phenotyping applies innovative technologies to exhaustively and more precisely examine the discrete components of a phenotype and goes beyond the information usually included in medical charts. This form of phenotyping is a critical component of more precise diagnostic capability and 3-dimensional facial analysis (3DFA) is a key technological enabler in this domain. In this paper, we examine the potential of 3DFA as a public health tool, by viewing it against the 10 essential public health services of the “public health wheel,” developed by the US Centers for Disease Control. This provides an illustrative framework to gage current and emergent applications of genomic technologies for implementing precision public health.

## Introduction

Rare diseases (RD) are increasingly recognized nationally (1) and globally as a public health priority (2, 3). While individually, RD have a low prevalence, it is estimated that the combined prevalence is between 6 and 8% of the population (2, 4). Most RD have a genetic association and are often severely debilitating, impair physical and mental abilities, and shorten life expectancy (5). These characteristics present clinical and public health challenges. These include the need for early and accurate diagnosis and for identifying emerging technologies to enhance the delivery of clinical and public health practices for affected individuals (1).

The RD community has collectively nominated timely accurate diagnosis and earlier intervention with improved therapeutic options as key issues (6). This context and this challenge also provide opportunities for innovation and creating new knowledge. One such opportunity for improved diagnosis and treatment is through the clarity that can be achieved with detailed analysis and representation of the phenotype of genetic and rare disorders. Broadly, a phenotype is the set of observable characteristics of an individual resulting from the interaction of a genotype with the environment; in medicine, it is used to describe some deviation from normal morphology, physiology, or behavior. Greater phenotypic clarity is being advanced through imaging, the use of standards for phenotypic description, and their combination. This “precision” or “deep” phenotyping affords medicine and science a unique opportunity to generate biological insights.

An emerging deep phenotyping application is 3-dimensional facial analysis (3DFA). In the RD domain, 3DFA has been investigated and is increasingly being implemented, primarily for diagnostic purposes (7–9). 3DFA is also being applied to monitor existing and novel therapies, an area in which it has a nascent role (10, 11). 3DFA involves the investigation of deeply precise 3D facial data that can be acquired with various facial imaging technologies and applied to deliver scientific insights. The technological innovations enabling 3DFA include advances in imaging hardware, analytical techniques, and the combination with other, e.g., text-based, advances.

Approaches such as 3DFA, and other forms of deep phenotyping, mean that RD are providing a fruitful domain for precision approaches to medicine and public health. This is highlighted by a series of targeted precision initiatives in multiple countries, including in the United States, programs based at the National Institutes of Health at the Centers for Mendelian Genetics and the Undiagnosed Diseases Program and Network (12, 13); in Japan, via its Agency for Medical Research and Development under its rare and intractable diseases pillar; and in Western Australia (WA), through a coordinated suite of initiatives being implemented under the *WA Rare Diseases Strategic Framework 2015–2018* (1, 9).

## Precision Technologies in Public Health

Precision public health has been defined as “the application and combination of new and existing technologies, which more precisely describe and analyze individuals and their environment over the life course, in order to tailor preventive interventions for at-risk groups and improve the overall health of the population.” Thus, precision public health complements and extends precision medicine’s focus by recognizing that precise interventions are needed at both the individual and population levels.

Herein, we outline the state of play of current and emergent 3DFA applications, specifically within a precision public health paradigm, and using congenital and rare disorders as an exemplar. As an illustrative framework, we use the 10 essential public health services of “the public health wheel” (14). This framework operationalizes the three core functions of public health, namely, assessment, policy-making, and assurance.

## Monitor Health Status to Identify and Solve Community Health Problems

Congenital anomalies are an important class of mainly RD accounting for 12–15% of people with RD and are also known as birth defects (15). The causes of these conditions can be divided into genetic (e.g., monogenic disorders), multifactorial (e.g., cleft palate), and environmental exposures [e.g., fetal alcohol syndrome (FAS)]. Congenital anomalies accounted for 732,000 disability-adjusted life years lost, in 2010, in Western Europe alone (16).

A considerable proportion of congenital anomalies are associated with facial dysmorphology (17), either through the presence of congenital anomalies in known syndromes with well-documented facial dysmorphology (e.g., cardiac anomalies in Noonan syndrome), or in the recurrent co-coding of individual congenital anomalies and facial dysmorphism in individuals (17). Furthermore, hundreds of disorders (18), which are collectively and variably described as “dysmorphic syndromes” or “developmental disorders,” have characteristic facies. In these instances, 3DFA has potential to contribute to the improved speed and accuracy of diagnosis for a sizeable proportion of the general population. This will contribute to more accurate epidemiological data, including more precise estimates of the incidence, prevalence, and burden of congenital disorders.

## Diagnose and Investigate Health Problems and Health Hazards in the Community

**Figure 1:**
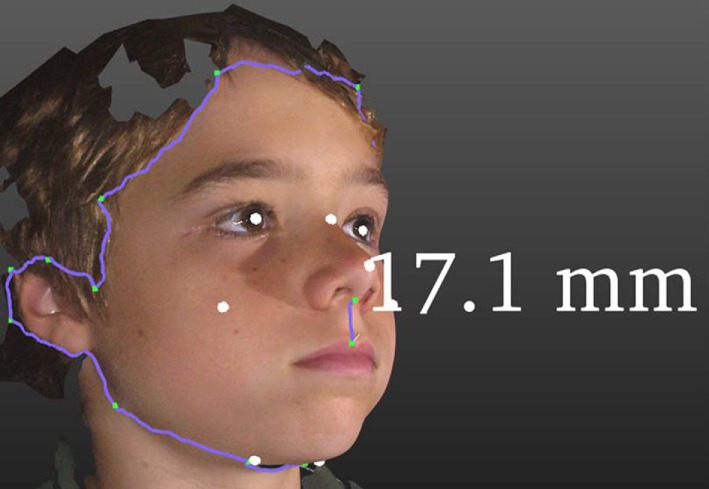
**Some functionality of 3DFA**. The purple line around the facial periphery demonstrates a cropping and facial segmentation tool. White dots are automated land marking. The vertical purple line demonstrates an application of the measuring tool, in this case showing a 17.1 mm philtral length.

**Figure 2:**
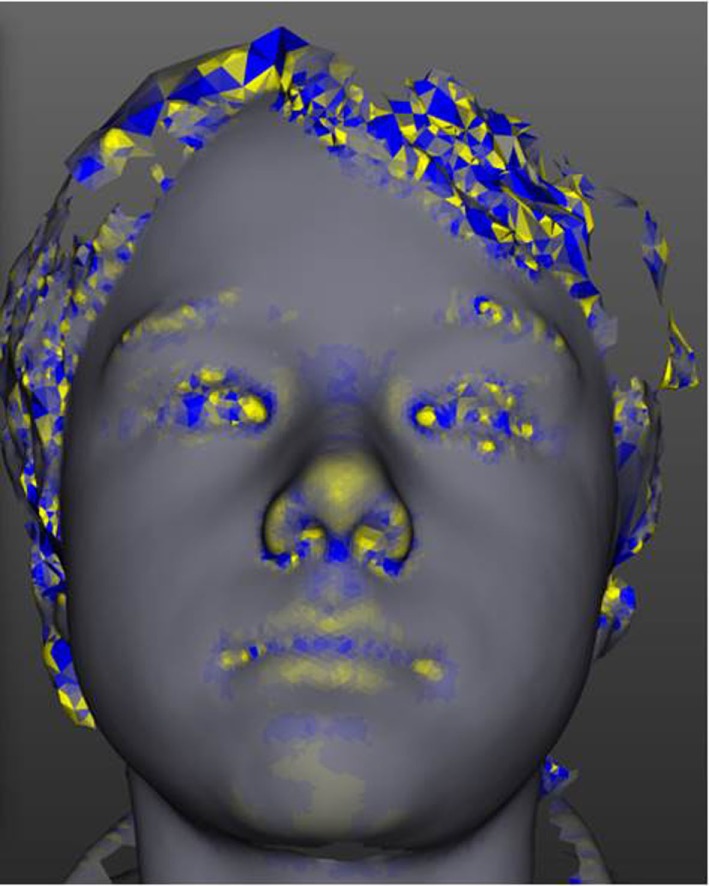
**The tool has modular analysis components**. This figure demonstrates one such module, curvature analysis, which is demonstrated in a yellow-blue color scale.

3-Dimensional facial analysis is being developed for deeply precise diagnostic applications across a broad range of typically rare conditions with well-established facial dysmorphic patterns (8), see Figures 1 and 2. Additionally, it is increasingly and objectively unlocking hitherto undetected, or underappreciated, facial diagnostic signatures (7). For example, speech delay is common in rare conditions, and in one study of kindergarten children, approximately 7% had language-specific impairments (19). A potential 3DFA application is using facial signatures as early predictors of language delay, either in those from the general population or in those at high familial risk, e.g., siblings of children with autism. The presence of a group of rare disorders, characterized by severe speech impairment and with overlapping facial features, collectively called Angelman-like syndromes (20), supports the possibility of using facial signatures to predict speech delay. There are also numerous other rare disorders that are associated with variable degrees of speech delay, e.g., Cornelia de Lange syndrome and biologically related disorders (21), that have characteristic, and overlapping facial phenotypes. It is likely that other children, with or without known syndromes, will have facial signatures that are indicative of speech delay that may offer a novel way for early screening for language delay to target early intervention.

## Inform, Educate, and Empower People about Health Issues

We all have a face. It is our unique expression of who we are, it reflects our life experiences and communicates our emotions to the world. From birth, our faces are a window to our being and our portal of interaction with our world. Our faces speak of the community from whence we came, and of the communities to which we belong, the ultimate expression of our connection as individuals. Our face is a canvas for the arts, a window for education, a living record of the diversity of the environment and our origins. Our face is also a biological billboard that advertises our physical and mental wellness, our aging, and our disease. We commonly say, “you look ill,” “you look well,” “you look in pain,” and we can, for instance, readily recognize a child with Down’s syndrome by their facial features. Objectively documenting and harnessing these facial clues that underlie common parlance and innate recognition capacities, can be used to inform, educate, and empower people for health.

A person who has a 3D image taken of their face can almost immediately see the computer-generated image. This recognizable and relatable image enables patients and their families to gain a new perspective of their health, or the health of a relative. Within WA, the technology has recently been used with primary school students who participated in a project to support equitable innovation for Aboriginal health. As the parameters of normal facial contours vary with ethnicity, it is important to compile reference scans for different ethnic groups. The children involved in this project were delighted to be able to view and manipulate their 3D facial images. “They especially loved being able to turn their faces upside down to look up their noses!” (22).

## Mobilize Community Partnerships and Action to Identify and Solve Health Problems

Projects focusing on the delivery of novel ways to diagnose and monitor rare disorders have been undertaken with the key support of patient advocacy organizations in Australia (e.g., Rare Voices Australia, Fabry Australia, Mucopolysachharide and Related Diseases Society Australia, Short Statured People’s Association of Australia) and internationally (e.g., International MPS Network, Costello Syndrome Family Association, CFC International). Similarly, projects focused on equitable health innovation to address RD have been developed and delivered in partnership with Aboriginal leaders, health workers, and communities. These projects have been important to the development of interest in and application of 3DFA.

The desire to address RD, together with the multifarious and crosscutting aspects of faces described above, has provided a unique vehicle for mobilizing community partnerships to engage in the identification and solution of health problems. A 2015 event organized as part of an ongoing platform for harmonizing translational research across premier hospitals, research institutes, government, and the community is an illustrative case (23). The “Faces of WA” event coalesced cross-sector interest in 3DFA applications from across science, arts, research, education, and data analytics communities.

## Develop Policies and Plans That Support Individual and Community Health Efforts

In 2015, WA released the state-wide *WA Rare Diseases Strategic Framework 2015–2018* (1), which includes, but is not limited, to the following objectives: build on existing services for RD diagnosis and screening; identify emerging technologies to enhance the delivery of health care for RD, including to rural and remote areas; engage with people living with RD, their carers and families; promote active participation of people living with RD with their health care; build epidemiology and health system evidence for RD by improving diagnosis and disease classification; and strengthen clinical and translational research in RD. The implementation of 3DFA contributes to most of these objectives.

An example of a disease-specific policy in WA is the *Fetal Alcohol Spectrum Disorders (FASD) Model of Care* (24). FASD has been prioritized as a public health issue in Australia and other countries, and a cohesive, multilevel, and community-focused suite of approaches is required to address this preventable disorder group. The *FASD Model of Care* identifies health care and public health prevention strategies as the most important means of reducing FASD. The implementation and use of 3DFA has the potential to address several of these recommendations, including but not limited to screening and early diagnosis. FAS is part of the of FASD continuum for which characteristic facial features are obligate for diagnosis. These facial characteristics are known to vary by ethnicity (25, 26), and there is a paucity of data on the Australian Aboriginal population. A pragmatic, but potentially imprecise, approach using African-American facial standards has been implemented for FAS diagnosis. Should these be unfit for purpose, epidemiological, and diagnostic data may be inaccurate with implications for targeted health and prevention strategies. Potentially, the ethnic variation of Aboriginal facial features could be more objectively addressed with the precision of currently available 3D approaches.

## Enforce Laws and Regulations That Protect Health and Ensure Safety

Clinicians and public health practitioners advocate for, review, evaluate, revise, educate, and enforce compliance with laws and regulations. This must include new and existing laws and regulations related to the use of genomic and other technologies, and the information generated from their use (27).

The genetic and genomic information about individuals, and that obtained from 3DFA, forms part of and expands the individual’s health information. It may also provide information about the individual’s relatives, which may be of interest to them, especially where prevention or treatment is available. Health professionals may inform the individual of how the genetic information relates to their relatives; however, confidentiality requirements prevent the disclosure of this information to the relatives, without the consent of the individual, except in specific circumstances outlined in relevant legislation and regulations. Internationally and variably, legislation has been enacted to regulate the collection, use, and disclosure of health information. In Australia, the 2006 amendments to *the Privacy Act 1988* allow for health practitioners to use or disclose an individual’s genetic information, without their consent, where there is a reasonable belief that doing so is necessary to lessen or prevent a serious threat to the life, health, or safety of their relatives. Irrespective of jurisdictional differences in legislation and its implementation, the principle remains that new phenotypic technologies that reveal indications of familial disease may have implications for life insurance, employment, and reproductive choices, so they need to conform with legislated codes for privacy protection, disclosure, and data sharing.

## Link People to Needed Personal Health Services and Assure the Provision of Health Care When Otherwise Unavailable

Linking people to needed services includes developing mechanisms to assure the provision of such services to marginalized and underserved populations. In relation to RD, three such population groups in WA are people living with long-standing undiagnosed conditions, those living in rural and remote areas, and Aboriginal Australians. Improved diagnostic services utilizing new genomic technologies and 3DFA are enabling more equitable access to services for these populations.

At the level of state-wide clinical practice, 3DFA has been implemented in services that improve population access to RD diagnostics. The Rare and Undiagnosed Diseases Diagnostic service at Genetic Services of WA (9) integrates genomic diagnostics into a state-wide clinical service that includes outreach clinics. The Undiagnosed Diseases Program Western Australia is a cross-disciplinary service provided within the local children’s hospital but also accessible to children across the state. These complementary programs aim to find diagnoses for those with long-standing, undiagnosed conditions.

Given the (increasing) transportability and reducing cost of 3D facial imaging systems, 3DFA is being implemented in remote outreach clinics by initially using a model of periodic deployment of a portable camera. Permanent placement of scanners in key regional locations is planned for the future. This is to facilitate point-of-care diagnostics, treatment, and monitoring and to enhance referral, i.e., by pairing submission of 3D images with text-based referrals and consultation processes.

## Assure Competent Public and Personal Health-Care Workforce

Facial gestalt is key to diagnosis for numerous genetic conditions (e.g., Velocardiofacial syndrome, Williams syndrome, Noonan syndrome) and non-genetic conditions [e.g., fetal valproate syndrome (FAS)]. Through the creation of tools that objectively determine facial patterns and unlock knowledge, diagnostic ability and workforce competency can be improved. The increasingly transportable nature of the approach also suits capacity building in remote regions; training in 3DFA could contribute to workforce development. Coupled with the non-invasive nature of 3DFA, this increasing portability, and the very nature of faces, it also provides a unique opportunity to engage with this new technology as a bridge to deeper engagement with other (e.g., genomic) technologies.

## Evaluate Effectiveness, Accessibility, and Quality of Personal and Population-Based Health Services

The value of clarifying a diagnosis is undeniable (28). Improved diagnostic certainty through the precision of 3DFA provides novel opportunities to evaluate health care, for instance, in assessing the diagnostic programs for rare genetic diseases through direct and objective comparison of facial phenotypic and molecular diagnostic approaches. Additionally, by improving certainty of the diagnosis of RD, one could more accurately assess interventions targeted to the reduction of the burden of these conditions. Given the marked disparity between the proportion of the population with RD and their combined health system costs, supporting the need for early diagnosis and intervention has the potential to drive cost savings across the health system (29).

3-Dimensional facial analysis is being used to monitor the effectiveness of drug therapy, which provides new avenues to assess drug response for both localized facial anomaly (10) and systemic disease (11). An example of 3DFA’s use to monitor drug response was an application in mucopolysaccharidosis type I (MPS I). This lysosomal storage disease is caused by the body’s inability to produce a specific enzyme, it expresses a pattern of progressive facial dysmorphology and it is treatable by drug therapy. 3DFA was used to monitor a child with MPS I undergoing treatment to demonstrate that the rate at which facial dysmorphology was advancing was reduced (11). 3DFA has also been used to monitor a child receiving a treatment (rapamycin) for a craniofacial anomaly. This child had extensive facial malformations and monitoring of their facial features using 3DFA showed a progressive improvement in their condition (10). In addition to observing treatment and disease progression in a standard clinical setting, 3DFA may also have a monitoring role in clinical trials.

## Research for New Insights and Innovative Solutions to Health Problems

Rare diseases are a hot bed for technological innovation and recurrently discoveries in RD have delivered innovations for common diseases (30). While 3DFA is yielding translational insights into innovations for diagnosis, treatment, and monitoring in the RD domain, it is also particularly suited to examining the overlap between rare and more common diseases. Notably, population level studies demonstrated that common genetic variations (polymorphisms) were associated with discrete patterns of facial variation. Notably, these facial signatures recapitulated the characteristic facies of the respective genetic syndrome due to rare genetic variation (pathogenic mutations). This highlights further evidence of the overlap between common and rare phenotypes with implications for possible reciprocal (rare-common) insights.

An example of a common disease that is poised for 3D facial translational research is obstructive sleep apnea (OSA). Through determining the facial signatures of OSA, 3DFA can be used as a complementary tool for OSA screening and classification. Again reflecting the potential for joint insights into rare and common diseases, OSA is a condition seen in RD, where it regularly has an earlier onset than in the general population (e.g., MPS syndromes).

A further promising area is face-to-text conversion. Conversion of a 3D facial image to standardized text-based descriptive terms known as human phenotype ontology (HPO) is an ideal way to achieve this. These standardized terms can be used computationally (i.e., are machine readable) and can then be used for report generation and for integration with text-based diagnostics. Face-to-text conversion has been performed for a limited subset of facial HPO terms (31). It needs to be extended to the full set and be further validated by human experts.

## Conclusion

3-Dimensional facial analysis is a prototypical precision public health tool that delivers non-invasive, non-irradiating, transportable, and community engaging deep phenotyping. It enables multisector applications that can be increasingly implemented across the spectrum of public health. It can be applied to individuals as well as for single RD. Finally, insights generated in RD could be investigated in more common diseases.

## Ethics Statement

The parents/legal guardians have consented to publish the image.

## Author Contributions

GB, HD, CM, and AB contributed equally to the coordination of content and drafting the manuscript. GB, NP, JG, and AH contributed expert content to the clinical genomics components. TW, HD, CM, CW, and AB contributed to the expert content on public health genomics. HV, LS, RP, SK, PH, and MR contributed to the informatics and 3-dimensional facial scanning content. GB, HD, JG, TW, and CW are chief investigators of the Australian National Health and Medical Research Council APP1055319, and LS is employed on this grant.

## Conflict of Interest Statement

The authors declare that the research was conducted in the absence of any commercial or financial relationships that could be construed as a potential conflict of interest.
